# Accelerated inflammatory aging in Alzheimer’s disease and its relation to amyloid, tau, and cognition

**DOI:** 10.1038/s41598-021-81705-7

**Published:** 2021-01-21

**Authors:** Nicholas C. Cullen, A nders Mälarstig, Erik Stomrud, Oskar Hansson, Niklas Mattsson-Carlgren

**Affiliations:** 1 Clinical Memory Research Unit, Department of Clinical Sciences, Faculty of Medicine, Lund University, Sölvegatan 19, BMC - C11, 223 62 Lund, Sweden; 2 Pfizer Worldwide Research & Development, Stockholm, Sweden; 3 Department of Medicine, Karolinska Institutet, Solna, Sweden; 4 Memory Clinic, Skåne University Hospital, Lund, Sweden; 5 Department of Neurology, Skåne University Hospital, Lund, Sweden; 6 Wallenberg Center for Molecular Medicine, Lund University, Lund, Sweden

**Keywords:** Neurology, Neurological disorders, Inflammation, Innate immunity

## Abstract

It is unclear how pathological aging of the inflammatory system relates to Alzheimer’s disease (AD). We tested whether age-related inflammatory changes in cerebrospinal fluid (CSF) and plasma exist across different stages of AD, and whether such changes related to AD pathology. Linear regression was first used model chronological age in amyloid-β negative, cognitively unimpaired individuals (Aβ− CU; n = 312) based on a collection of 73 inflammatory proteins measured in both CSF and plasma. Fitted models were then applied on protein levels from Aβ+ individuals with mild cognitive impairment (Aβ+ MCI; n = 150) or Alzheimer’s disease dementia (Aβ+ AD; n = 139) to test whether the age predicted from proteins alone (“inflammatory age”) differed significantly from true chronological age. Aβ− individuals with subjective cognitive decline (Aβ− SCD; n = 125) or MCI (Aβ− MCI; n = 104) were used as an independent contrast group. The difference between inflammatory age and chronological age (*InflammAGE* score) was then assessed in relation to core AD biomarkers of amyloid, tau, and cognition. Both CSF and plasma inflammatory proteins were significantly associated with age in Aβ− CU individuals, with CSF-based proteins predicting chronological age better than plasma-based counterparts. Meanwhile, the Aβ− SCD and validation Aβ− CU groups were not characterized by significant inflammatory aging, while there was increased inflammatory aging in Aβ− MCI patients for CSF but not plasma inflammatory markers. Both CSF and plasma inflammatory changes were seen in the Aβ+ MCI and Aβ+ AD groups, with varying degrees of change compared to Aβ− CU and Aβ− SCD groups. Finally, CSF inflammatory changes were highly correlated with amyloid, tau, general neurodegeneration, and cognition, while plasma changes were mostly associated with amyloid and cognition. Inflammatory pathways change during aging and are specifically altered in AD, tracking closely with pathological hallmarks. These results have implications for tracking AD progression and for suggesting possible pathways for drug targeting.

## Introduction

The term “brain age” has emerged in recent years to describe the phenomenon whereby individuals’ brain morphology is highly predictive of chronological age. Using this framework, studies have shown that diseases of the brain exacerabate the normal trajectory of brain aging such that individuas with neurodegenerative disease have a brain structure more akin to a significantly older, healthy individual^1,2^. Alzheimer’s disease (AD), the most common form of dementia, is characterized in particular by significant brain atrophy beyond what is expected during normal aging, and the discrepancy between biological-based brain age and chronologsical age can both predict future outcomes in individuals at an early stage of the disease, as well as aid in differential diagnosi^3–5^.

This brain age paradigm can also be adapted to other types of data in order to describe other types of biological aging^6^. With the increased availability of large-scale proteomic screening tools, for instance, it is now prudent to investigate proteomic aging in the context of AD. Inflammatory proteomics is particularly well-suited to contribute to our understanding of AD by identifying subject-specific inflammatory risk patterns^7^. The relevance of inflammatory aging in AD is supported by observations of age-related changes in inflammatory pathways and the established connection between increased inflammatory response and AD-related pathophysiological changes^8,9^.

Inflammation has been shown to be highly coupled with AD progression, but the role of innate immunity in AD is largely unexplored from a clinical perspective^10^. Chronic inflammation has been implicated in the onset and progression of AD, and a causative role for inflammatory pathways in AD is supported by genetic evidence^11,12^. However, most clinical studies rely on a single, broad measure of inflammation and thus it remains unclear which inflammatory pathways in particular are altered by AD progression beyond what is expected in normal aging.

In the current study, we explored accelerated aging of the cerebrospinal fluid (CSF) and plasma inflammatory proteome in both preclinical and clinical stages of AD in a large cohort study of 859 individuals using 73 inflammatory proteins measured in both CSF and plasma. Our main hypothesis was that inflammatory protein levels would be significantly associated with chronological age in healthy individuals without biomarker evidence of AD, and that individuals who find themselves at various stages in the AD continuum would be characterized by biologically older inflammatory proteomes. Furthermore, we hypothesized that key hallmarks of AD, including CSF measures of amyloid-β (Aβ) and tau along with cognitive decline, would be related to the actual magnitude of inflammatory aging at an individual level.

## Methods

### Study population

The study population came from the Swedish BioFINDER (Biomarkers For Identifying Neurodegenerative Disorders Early and Reliably) study—see biofinder.se for more details. Individuals included in the current analysis include Aβ− cognitively unimpaired (Aβ− CU; n = 312), Aβ− subjective cognitive decline (Aβ− SCD; n = 125), Aβ− mild cognitive impairment (Aβ− MCI; n = 104), Aβ+ mild cognitive impairment (Aβ+ MCI; n = 150), and Aβ+ AD dementia (Aβ+ AD; n = 139) individuals. A second group of Aβ− CU individuals (n = 29) independently recruited as controls in a Parkinson’s disease sub-study of BioFINDER served to validate the original models, while individuals in the Aβ− SCD and Aβ− MCI groups served as additional groups to which Aβ+ MCI and Aβ+ AD groups were compared.

CU subjects were originally enrolled from the population-based EPIC cohort and the Nomas 3 study^13^. The inclusion criteria were the following: age greater than or equal to 60 years old, Mini-Mental State Examination (MMSE) score at or above 28 during screening and at or above 27 at baseline visit, fluent in Swedish. MCI individuals were recruited consecutively and were thoroughly assessed by physicians with special competence in dementia disorders. The inclusion criteria were: referred to a memory clinic due to possible cognitive impairment, not fulfilling the criteria for dementia, MMSE score 24–30, at least 60 years old, and fluent in Swedish^14^. AD individuals were also recruited consecutively and fulfilled the NIA-AA criteria for probable AD^15^. Dichotomized Aβ status was determined using a pre-defined cutoff of the CSF Aβ42/40 ratio (assay described in more detail below)^16^.

The Regional Ethics Committee in Lund, Sweden, approved the study and all subjects gave written informed consent. All procedures were carried out in accordance with relevant guidelines.

### Protein and biomarker quantification

Inflammatory protein concentrations were quantified using the highly sensitive and specific Proseek multiplex immunoassay, developed by Olink Proteomics (Uppsala, Sweden)^17^. A total of 92 proteins (73 of which were used due to having available inflammatory process classification, see below) from the Inflammation I multiplex panel (see https://www.olink.com/products/inflammation/ for more details) were measured in both CSF and human plasma. Protein measurement was conducted using Proximity Extension Assay (PEA) technology following the manufacturer’s protocol and has been described in detail previously^17^. Protein quantities were produced as normalized protein expression (NPX) values on the log2 scale and measurement values below the assay detection limit were not included in the analysis. No imputation was performed on missing data and thus only participants with all available measurements were included in the analysis.

All proteins in the panel were further classified a priori according to biological process/pathway, disease area, tissue expression, and protein class. The 11 functional pathway classifications used in the present study were as follows: (1) apoptotic process, (2) cell activation involved in immune response, (3) cell adhesion, (4) cellular response to cytokine stimulus, (5) chemotaxis, (6) extracellular matrix organization, (7) inflammatory response, (8) MAPK cascade, (9) regulation of immune response, (10) response to hypoxia, and (11) secretion. Classification of a given protein into one of these 11 inflammatory process occured based on documention for that protein which was available in widely used public-access bioinformatic databases—including Uniprot, Human Protein Atlas, Gene Ontology (GO), and DisGeNET. Nearly all proteins belonged to more than one functional group, with proteins belonging on average to 3.6 different processes. A full list of which proteins belong to each inflammatory pathway is found in Table S1.

As a sensitivity analysis and to avoid potential issues with a priori classification, all proteins were included in a single model and the set of proteins which best predicted age in the Aβ− CU group (“data-driven cluster”) were selected using LASSO regression (described in more detail below).

In terms of AD-related biomarkers, CSF levels of the Aβ42/Aβ40 (representing amyloid accumulation in the brain), tau phosphorylated at threonine 181 (p-tau; representing tau accumulation in brain), and total tau (t-tau; representing neurodegeneration in the brain) were measured for all participants using the Euroimmun platform^18^. Cognitive measures included in the analysis were the Mini-Mental State Examination (MMSE) and the Clinical Dementia Rating Scale-Sum of Boxes (CDR-SB), both of which have established relevance for diagnosis and tracking of disease progression, along with use as endpoints in AD clinical trials^19^.

### Statistical analysis

First, linear regression was used to establish the relationship between CSF or plasma inflammatory proteins and chronological age in Aβ− CU individuals. All 73 inflammatory proteins were included as predictors in the model along with adjustment for sex, education, and mean protein level. The response variable was true chronological age. The relationship between the overall model and chronological age was assessed for statistical significance using the F-test.

Next, age was predicted for each individual in the other groups—Aβ− CU, Aβ− SCD, Aβ− MCI, Aβ+ MCI and Aβ+ AD—by supplying each individual’s inflammatory protein values to the previously fitted models. The difference between age predicted by the fitted regression model (i.e. inflammatory age) and chronological age was then calculated to determine an *InflammAGE* score. An *InflammAGE* score of + 5 for a 65 year old individual, for example, means that this individual’s inflammatory proteome is representative of an amyloid-negative, cognitive unimpaired individual who is 70 years old. Significantly increased *InflammAGE* scores compared to zero (indicating significant inflammatory aging compared to what is expected in normal aging) was then evaluated across group using analysis of variance (ANOVA) and between each groups using pairwise t-tests.

The association between *InflammAGE* score and AD-related biomarkers in CSF (Aβ42, t-tau, and p-tau) and cognition (MMSE, CDR-SB) was tested using linear regression with the *InflammAGE* score as predictor and each biomarker separately as dependent variable, with adjustment for chronological age, sex, education, clinical diagnosis, and amyloid status.

This analysis was performed on the proteins for each of the 11 inflammatory processes and a sensitivity analysis was performed using sparse LASSO regression to select the optimal set of proteins from the entire group (“data-driven cluster”). All statistical modelling was performed in the R (v4.0.0) programming language^20^. All tests were two-sided with a significant level set to p < 0.05 and adjustment for multiple comparisons using the Benjamini–Hochberg procedure where appropriate.

### Ethics approval and consent to participate

Written informed consent was received from all individuals prior to participation in the BioFINDER study. Ethics approval was received by the institutional review board at Lund University.

## Results

### Cohort characteristics

 regards to cognition, the Aβ+ AD group had significantly lower MMSE scores compared to Aβ+ MCI (P < 0.0001), which in turn had significantly lower MMSE scores compared to both Aβ− SCD and Aβ− CU (P < 0.0001 for both). The Aβ− SCD group also had slightly lower MMSE scores compared to Aβ− CU (P = 0.001). With regards to age, there was no difference in age between Aβ+ MCI and Aβ+ AD or between Aβ− groups and Aβ+ MCI, although the Aβ+ AD group was significantly older than the other Aβ− groups (P < 0.001). Additionally, the main Aβ− CU cohort was slightly older than the validation Aβ− CU cohort, although this difference was not significant. With regards to sex, there were more males in the Aβ+ MCI (53.3%) and Aβ+ SCD (56.9%) groups than in Aβ− CU (33.1%) and Aβ+ AD (37.1%) groups, thereby making it necessary to include sex as a covariate in all statistical models. Demographic information is further summarized in Table 1.

**Table 1:** Cohort demographics.

Clinical diagnosis	Aβ status	N	Age	Male (%)	CDRSB	MMSE
CU	**–**	312	71.6 ± 5.2	38.5	0.0 ± 0.0	29.1 ± 0.9
CU—validation	**–**	29	68.7 ± 5.3	45.8	0.0 ± 0.0	28.5 ± 1.3
SCD	**–**	125	69.5 ± 5.3	44.4	0.5 ± 0.7	28.7 ± 1.3
MCI	**–**	104	69.3 ± 5.6	67.8	1.3 ± 0.9	27.3 ± 2.0
	**+**	150	72.3 ± 4.9	53.3	1.5 ± 0.9	26.8 ± 1.7
AD	**+**	139	75.5 ± 5.8	37.1	NA	21.2 ± 3.9

All continuous values are reported as mean and standard deviation. Amyloid status was measured using the CSF Aβ42/40 ratio. The Aβ- CU group was used to fit the proteome-age regression models.

*CU* Cognitively unimpaired, *SCD* subjective cognitive decline, *MCI* mild cognitive impairment, *AD* Alzheimer’s disease dementia.

### Inflammatory proteins change significantly during healthy aging

**Figure 1:**
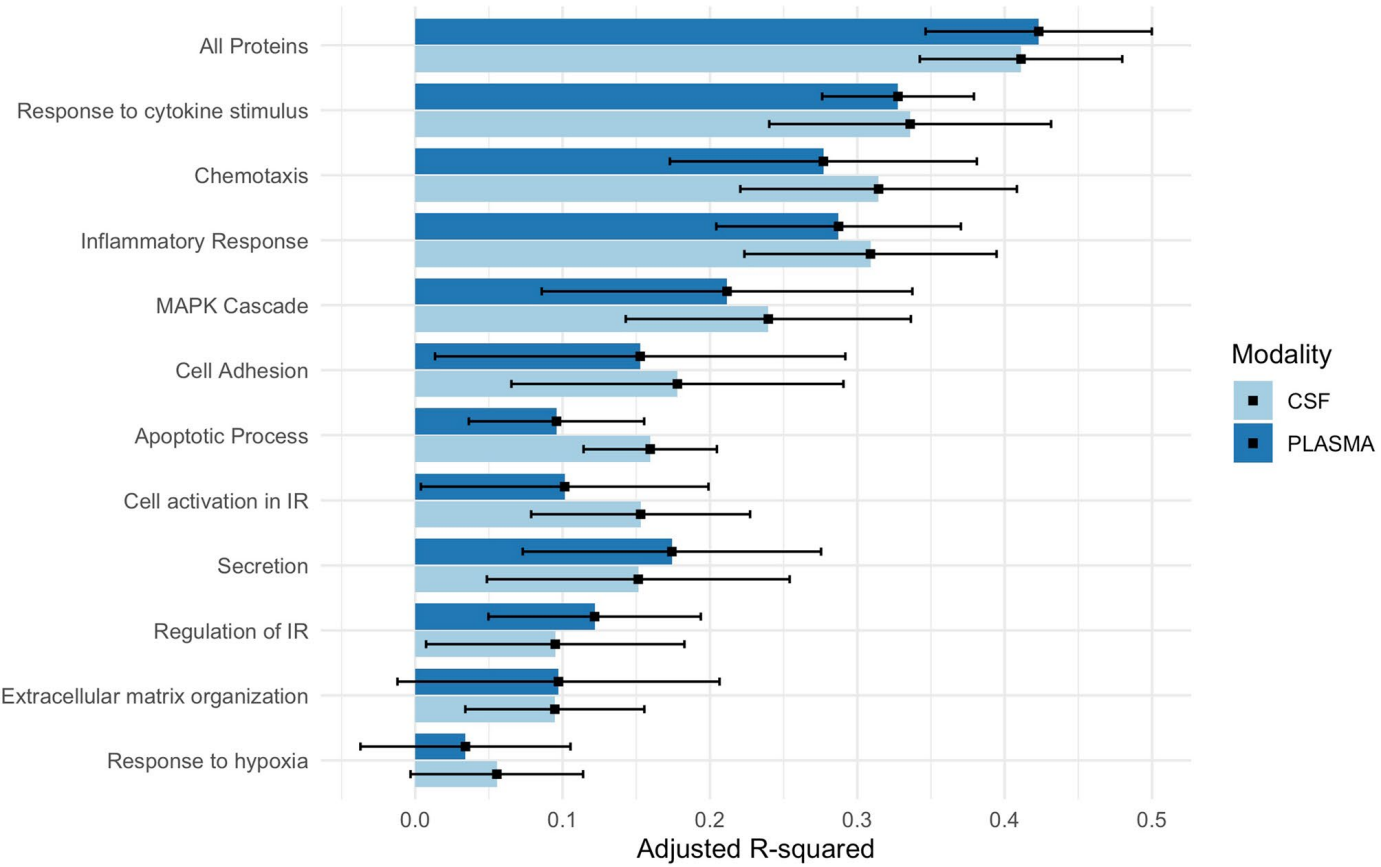
Predicting age from inflammatory proteins in Aβ-CU individuals. This figure shows the variance explained by linear regression models used to predict chronological age in amyloid-negative, cognitively unimpaired individuals from CSF and plasma proteins grouped into a range of inflammatory pathways.

CSF inflammatory proteins accurately predicted age in Aβ− CU individuals better than expected by chance for all inflammatory pathways besides “response to hypoxia” (P < 0.0001 for all). The variance explained (R^2^) by the CSF-based models ranged from 6 to 41% across inflammatory pathways (Fig. 1; Table S1), with the pathways most associated with age being “response to cytokine stimulus” (R^2^ = 0.34), “inflammatory response” (R^2^ = 0.31), and “chemotaxis” (R^2^ = 0.31). The data-driven protein cluster had the closest association with age (R^2^ = 0.41; see Table S3 for included proteins).

In plasma, chronological age was accurately predicted in the Aβ− CU group by all inflammatory pathways excepted for “response to hypoxia” and “extracellular matrix organization,” and the variance explained by age-prediction models ranged from 3 to 42% (Fig. 1; Table S1). Here, the pathways which explained the most variability in chronological age were again “response to cytokine stimulus” (R2 = 0.33), “inflammatory response” (R2 = 0.29), and “chemotaxis” (R2 = 0.29). Again, the data-driven protein cluster had the closest association with age (R^2^ = 0.42; see Table S3 for included proteins).

### InflammAGE models were validated in independent Aβ− groups

**Figure 2:**
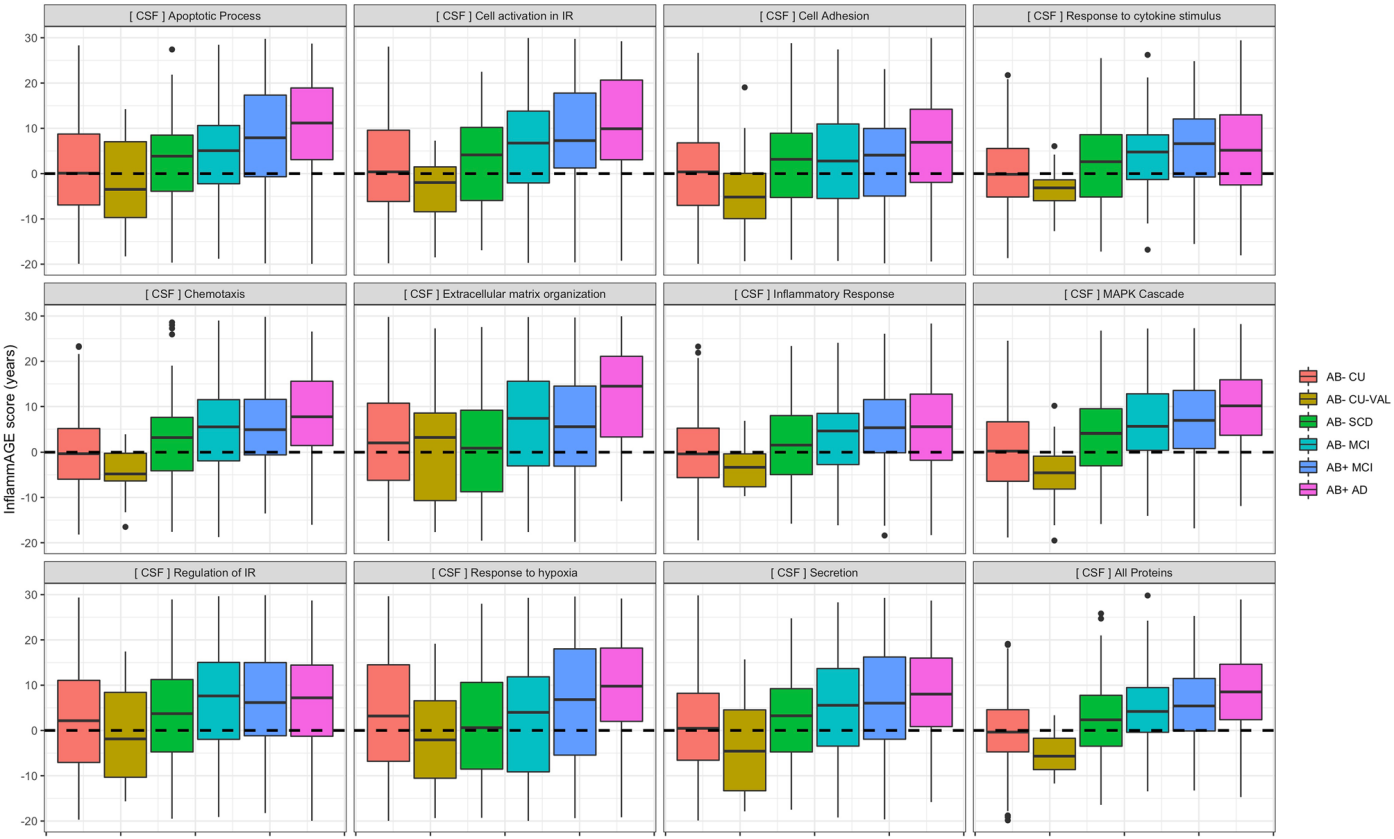
CSF-based proteomic aging across inflammatory pathways. This figure shows the distribution of CSF-based inflammatory age scores (i.e. the age predicted for each individual from inflammatory proteins alone) across groups and inflammatory processes. The dotted line at zero indicates that the age predicted from an individual’s protein levels is the same as that individual’s chronological age—indicating an inflammatory age which is normal. Values above the dotted line indicate that an individual’s inflammatory proteome is more representative of a chronologically older individual.

**Figure 3:**
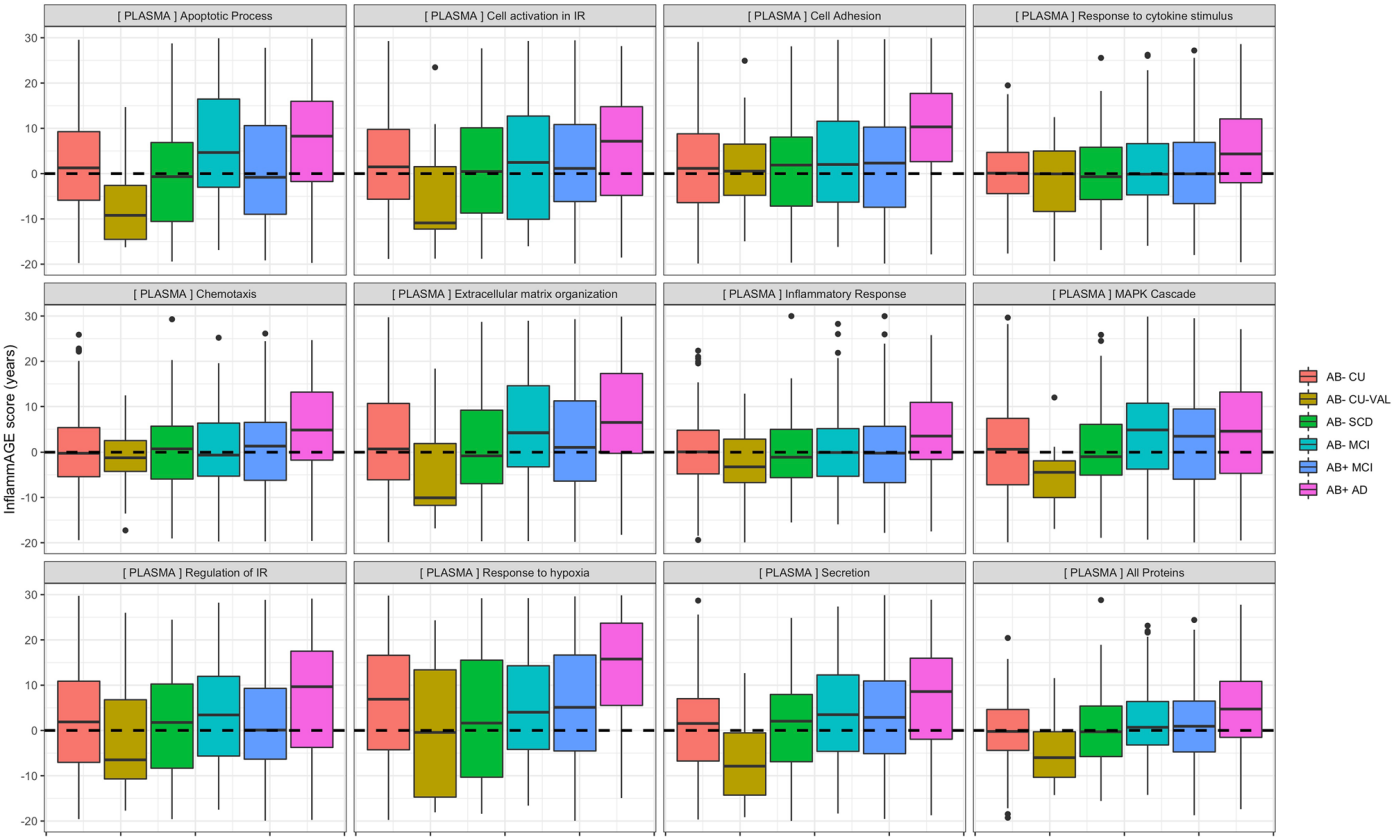
Plasma-based proteomic aging across inflammatory pathways. This figure shows the distribution of plasma-based inflammatory age scores (i.e. the age predicted for each individual from inflammatory proteins alone) across groups and inflammatory processes. The dotted line at zero indicates that the age predicted from an individual’s protein levels is the same as that individual’s chronological age—indicating an inflammatory age which is normal. Values above the dotted line indicate that an individual’s inflammatory proteome is more representative of a chronologically older individual.

Validation of regression models occurred in an independently recruited Aβ− CU group, where the goal was to show that there was not any significant inflammatory aging in this Aβ− CU validation group. Here, we showed that *InflammAGE* scores were not significantly greater than zero for any inflammatory pathways in either CSF or plasma, indicating that the validation Aβ− CU group did not have significantly older inflammatory proteomes compared to the main Aβ− CU group (Figs. 2, 3; Table S4). In fact, there were multiple processes for which the validation group actually had significantly lower *InflammAGE* scores, indicating that the validation group was actually characterized by healthier-than-expected inflammatory proteomes.

Validating *InflammAGE* scores in the Aβ− SCD group—where again, we expected to see no increased inflammatory aging—to those in the Aβ− CU group indicated significantly increased CSF-based *InflammAGE* scores only for “MAPK cascade” and the data-driven cluster, while there were no pathways for which plasma-based *InflammAGE* scores were increased in Aβ− SCD compared to Aβ− CU (Figs. 2, 3; Table S4).

Finally, in the Aβ− MCI group we observed significantly increased *InflammAGE* scores compared to Aβ− CU for all inflammatory pathways in CSF besides “regulation of immune response” and “response to hypoxia” pathways. In plasma such an increase was found only for “MAPK cascade” and “secretion” pathways (Figs. 2, 3; Table S4).

### Elevated inflammAGE scores were observed in Aβ+ MCI and AD groups

Next, we tested which pathways were altered in Aβ+ MCI and Aβ+ AD groups compared to both Aβ− CU and to Aβ− MCI groups.

Here, found that in the Aβ+ MCI group all inflammatory pathways besides “cell adhesion” were altered compared to Aβ− CU, while only the “apoptotic process” pathway was altered compared to Aβ− MCI (Figs. 2, 3; Table S5). In the Aβ+ AD group, all inflammatory pathways were altered compared to Aβ− CU, while “apoptotic process”, “extracellular matrix organization”, “response to hypoxia”, and the data-driven cluster were altered also compared to Aβ− MCI (Figs. 2, 3; Table S5).

For plasma-based inflammatory proteins, no pathways in the Aβ+ MCI group were altered compared to the Aβ− CU group or compared to the Aβ− MCI group (Figs. 2, 3; Table S5). For the Aβ+ AD group, however, all pathways were altered compared to the Aβ− CU group and “cell adhesion”, “regulation of immune response”, and “response to hypoxia” pathways were altered compared to the Aβ− MCI group (Figs. 2, 3; Table S5).

### InflammAGE score is associated with pathological hallmarks of AD

**Figure 4:**
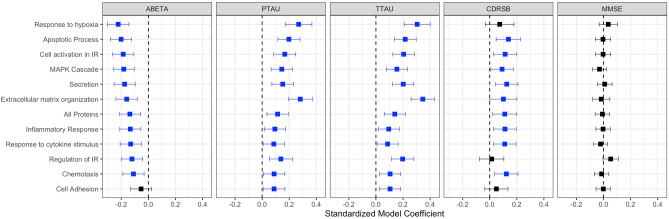
Association between CSF-based proteomic aging and core AD biomarkers. This figure shows the association between CSF-based *InflammAGE* scores (i.e. the difference between an individual’s inflammatory age and their chronological age) and various core biomarkers of AD—amyloid accumulation, tau accumulation, general neurodegeneration, and cognitive decline.

For CSF-based inflammatory proteins, *InflammAGE* scores in all pathways besides “cell adhesion” were related to CSF Aβ42 levels. *InflammAGE* scores in all pathways were also associated with CSF p-tau and t-tau levels. *InflammAGE* scores in all pathways besides “response to hypoxia”, “regulation of immune response”, and “cell adhesion” were associated with CDRSB, while no pathways were associated with MMSE (Fig. 4). The data-driven cluster in CSF was associated with Aβ42, p-tau, t-tau, and CDRSB.

**Figure 5:**
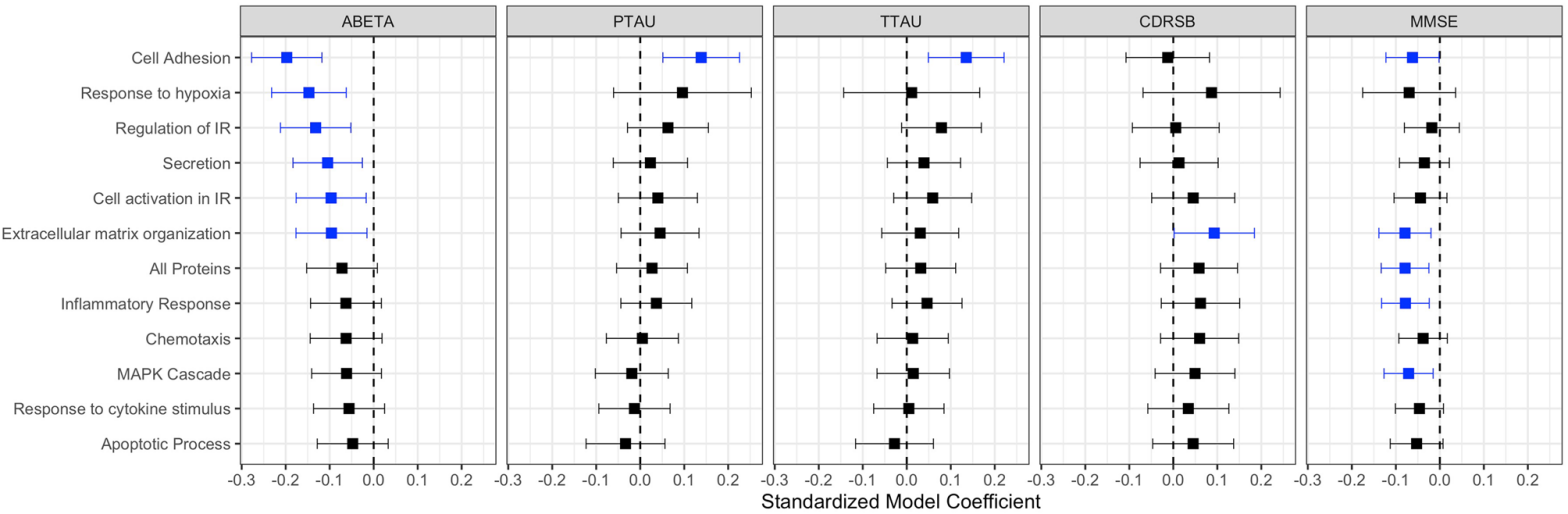
Association between Plasma-based proteomic aging and core AD biomarkers. This figure shows the association between plasma-based *InflammAGE* scores (i.e. the difference between an individual’s inflammatory age and their chronological age) and various core biomarkers of AD—amyloid accumulation, tau accumulation, general neurodegeneration, and cognitive decline.

For plasma-based inflammatory proteins, *InflammAGE* scores in “cell adhesion”, “response to hypoxia”, “regulation of immune response”, “secretion”, “cell activation in immune response”, and “extracellular matrix organization” were associated with CSF Aβ42 levels, while *InflammAGE* scores in “cell adhesion” only were associated with CSF p-tau and t-tau levels. Also, only *InflammAGE* scores in the “extracellular matrix organization” pathway was associated with CDRSB, while *InflammAGE* scores in “cell adhesion”, “extracellular matrix organization”, “inflammatory response”, and “MAPK cascade” pathways were associated with MMSE (Fig. 5). The data-driven cluster in plasma was associated only with MMSE.

## Discussion

In the present study, we measured 73 inflammatory proteins in both CSF and plasma in a large clinical cohort in order to investigate inflammatory pathway changes in AD. Our finding that both CSF and plasma proteins were highly predictive of age in Aβ− CU individuals adds to the established evidence that the innate immune system changes even during healthy aging^21^. From this basis, we showed that the AD continuum is characterized by accelerated biological aging of the innate immune system such that MCI and AD patients have inflammatory proteomes which are akin to healthy individuals who are significantly older. This finding is in line with similar biological aging studies of AD carried out using structural brain imaging (i.e. brain age), for example^3^. Our results were carefully controlled for both sex and educational attainment; education was specifically accounted for due to its known association with dementia and because it may even be considered as a proxy for socioeconomic factors that affect baseline cognitive capacity^22^.

Importantly, the abnormal inflammatory aging we observed in the AD continuum is differentially expressed across specific inflammatory pathways and can even differ depending on whether proteins are measured in CSF or plasma. For instance, our results showing that plasma-based inflammatory aging was elevated in AD patients compared to Aβ− MCI patients suggest that cross-sectional inflammatory levels as measured in plasma may be more AD-specific at a given point in time compared to those measured in CSF. Observing longitudinal changes was unfortunately outside the scope of our current work. Meanwhile, since CSF-based inflammatory aging correlated strongly with core AD biomarkers and predicted chronological age better than plasma in the main Aβ− CU group, CSF inflammatory proteins likely track more closely with those brain-based inflammatory changes which occur during normal aging. This finding may be explained by higher analytical variability in plasma measurements or the fact that plasma inflammatory protein concentrations are influenced by more abundant proteins found in the periphery^23^. Still, inflammatory proteins measured in plasma are theorized to be a potential source for detecting Alzheimer-related changes based on the idea that AD has a systemic metabolic state which can be observed in the periphery combined with recent improvements in plasma-based assays^24,25^. In any case, we hypothesize that plasma and CSF inflammatory proteins in general measure different but at least partially overlapping processes, although the task of identifying where this border lies still remains. By fitting the models in our study to highlight those proteins most closely associated with chronological age in healthy individuals, however, we hoped to thereby maximize the overlap between plasma and CSF.

We further found that increased CSF inflammatory levels were observed in Aβ− SCD and Aβ− MCI groups for numerous processes, while such was not the case for plasma. Meanwhile‚ increased plasma-based inflammatory protein levels for various processes were often observed only in the AD group. This may also indicate that CSF inflammatory proteins proceed those seen in respective plasma proteins—a phenomenon which is also seen for core AD biomarkers^26^.

One major assumption of our analysis is that healthy individuals will eventually have similar inflammatory profiles to AD patients as they age normally. However, it is difficult in the current study to separate age- and disease-related inflammation and thus it may be the case that AD patients are simply on a parallel trajectory of inflammatory change due to the disease. Indeed, there are profound differences in both causes and effects between aging-related and Alzheimer-related inflammatory changes^27,28^. However, the fact that our inflammatory aging models were fit based only on data from healthy individuals across a wide age range and without amyloid accumulation means that the changes we observed in AD patients are those most likely to be normal age-related. An AD patient predicted by our models to have an “older” inflammatory age should therefore be expected have an altered inflammatory profile as it relates to those proteins which change naturally during normal aging.

Our findings have strong implications for the use of inflammatory proteins as biomarkers to identify or track the progression of AD, particularly as plasma assays for the core AD biomarkers appear to have strong performance in both prognostic and clinical trial scenarios^29,30^. In terms of drug discovery, our results point out a group of inflammatory pathways whose further study could illuminate the role of inflammation in AD pathogenesis. With regards to study limitations, we acknowledge that having an “older” inflammatory proteome can be explained by numerous factors. Previous studies suggest that the innate immune system decreases in effectiveness and is increasingly activated with age and thus this may be one suitable interpretation^21,31^. Methodologically, this study is limited by its cross-sectional in design and the fact that the Olink panel only allows for relative quantification of proteins rather than absolute quantification. Understanding how the inflammatory proteome actually changes in a single individual is therefore not possible within the current analysis. It is also difficult to use such cross-sectional clinical studies to determine whether abnormal inflammatory activation is a driver of AD pathology or simply a side-effect of the disease process^12^. Additionally, we did not focus on individual inflammatory proteins but rather turned our attention to higher-level inflammatory processes, thus leaving some room for interpretation as to what inflammatory pathways are being specifically targeted in our analysis.

Still, the large number of participants in our study combined with core AD biomarker data allowing for an objective, biological approach to patient classification adds great reliability to our results^32^. Moreover, the biological aging paradigm we employed is particularly well-suited for studies with large control groups such as ours, where a reliable trajectory can be established with regard to how a biological process changes during normal aging^6^. Our findings can be used as the basis for more detailed studies of inflammatory changes in AD from a fluid biomarker perspective. In the context of existing work on examing inflammatory changes in AD, our study focuses more on pathway-level changes instead of identifying individually altered single proteins as is commonly done^33^. The strong influence of cytokines and chemokines in our results is in strong agreement with previous studies which suggest a potential protective role such proteins upregulated during AD^34,35^. It is also possible that our analysis targeted pathways related to oxidative stress which are known to be altered in AD^36^. Future work may also involve investigating whether biomarkers of inflammatory alterations in AD provide additional value to tracking AD progression on top of the existing core AD biomarkers of amyloid, tau, and neurodegeneration.

## Conclusions

Our study demonstrates that inflammatory aging is readily measured in AD and relates directly to pathophysiological changes during the disease process. Our results have strong implications for targeting inflammatory processes for treating AD and for tracking AD progression using inflammatory markers. These findings also suggest that a more nuanced view of inflammation in the context of AD is warranted.

## Supplementary Information

Supplementary Information.

## Data Availability

Data is available in event of collaboration. All programming code used in analysis is available upon request.

## Abbreviations

MCI: Mild cognitive impairment
AD: Alzheimer’s disease
CDR-SB: Clinical dementia rating-sum of boxes
MMSE: Mini-mental state examination
CSF: Cerebrospinal fluid
Aβ: Beta-amyloid protein
t-tau: Total tau
p-tau: Phosphorylated tau
