# Supplementary material for: Accelerated inflammatory aging in Alzheimer’s disease and its relation to amyloid, tau, and cognition
Source: Sci Rep. 2021 Jan 21;11:1965. doi: 10.1038/s41598-021-81705-7 (PMC7820414; doi:10.1038/s41598-021-81705-7)
Supplement: Supplementary file 1 — Supplementary Information. [file 41598_2021_81705_MOESM1_ESM.docx]

SUPPLEMENTARY MATERIALS

**Accelerated inflammatory aging in Alzheimer’s disease and its relation to amyloid, tau, and cognition**

Nicholas C. Cullen^1^, Anders Mälarstig^2,3^, Erik Stomrud^1^, Oskar Hansson^1^, Niklas Mattsson-Carlgren^1,4,5^

^1^Clinical Memory Research Unit, Department of Clincal Sciences, Faculty of Medicine, Lund University, Lund, Sweden

^2^Pfizer Worldwide Research & Development, Cambridge, Massachusetts, 02139, United States.

^3^Department of Medicine, Karolinska Institutet, Sweden

^4^Department of Neurology, Skåne University Hospital, Sweden

^5^Wallenberg Center for Molecular Medicine, Lund University, Lund, Sweden

**Table S1: List of proteins across inflammatory processes**

| **Inflammatory** **Process** | **Proteins** |
| --- | --- |
| **Apoptotic Process** | STAMBP, IL-33, ADA, LAP TGF-beta-1, Beta-NGF, TNFB, TNF, IFN-gamma, IL-1 alpha, IL-4, IL6, CD5, CCL3 |
| **Cell activation in IR** | uPA, ADA, LAP TGF-beta-1, IFN-gamma, IL-4, IL6, CXCL1, CCL3, IL10, CD40, IL-12B, IL-13, IL-2, EN-RAGE , SLAMF1, IL-18R1, IL-18, CCL19, CD244 |
| **Cell Adhesion** | CCL25, TNFSF14 , uPA, ADA, LAP TGF-beta-1, TNF, IFN-gamma, CD8A, IL-4, IL6, CD5, CSF-1, IL-8, IL-7, MCP-1, VEGF-A, IL10, IL-12B, CD6, IL-2, CX3CL1 , IL-18R1, IL-18, CASP-8 , CCL19, CCL28, PD-L1, CCL4 , SCF, CCL11, SLAMF1 |
| **Response to cytokine stimulus** | CXCL11, TRANCE, CCL25, Ligand, TNFSF14 , IL-33, LAP TGF-beta-1, TNFB, TNF, IFN-gamma, IL-1 alpha, CXCL10 , MMP-1, IL-4, IL5, IL6, CXCL1, CSF-1, IL-8, CCL3, IL-7, CCL4 , MCP-1, OSM, HGF, IL-2RB, LIF, VEGF-A, IL10, CD40, IL-12B, IL-13, LIF-R, CXCL5 , Flt3L, CCL11, CCL23, IL-2, CX3CL1 , CCL20, MCP-2, MCP-3, CXCL6, TNFRSF9, CXCL9 , IL-10RB, IL-24, IL-15RA, IL-18R1, IL-10RA, IL-18, CASP-8 , IL-17A, IL-22 RA1, TSLP, MCP-4, CCL19, FGF-23, IL-20, IL-17C, IL-20RA |
| **Chemotaxis** | TRANCE, CCL25, TNFSF14 , LAP TGF-beta-1, CXCL10 , IL6, CXCL1, CSF-1, IL-8, CCL3, CCL4 , MCP-1, HGF, VEGF-A, NT-3, IL10, GDNF, CXCL5 , CCL11, CCL23, CX3CL1 , CCL20, MCP-2, MCP-3, CXCL6, EN-RAGE , CXCL9 , ARTN, MCP-4, CCL19, NRTN, CCL28, uPA |
| **Extracellular matrix organization** | MMP-1, MMP-10, OPG, TNF |
| **Inflammatory Response** | IL-33, ADA, LAP TGF-beta-1, TNFB, TNF, IL-1 alpha, IL6, CCL3, OSM, HGF, IL10, IL-12B, CD6, CCL11, IL-2, CX3CL1 , EN-RAGE , IL-18, IL-17A, TSLP, IL-20, OPG, CXCL11, CCL25, CXCL10 , IL5, CXCL1, CSF-1, IL-8, CCL4 , MCP-1, CD40, IL-13, CXCL5 , CCL23, CCL20, MCP-2, MCP-3, CXCL6, TNFRSF9, CXCL9 , IL-10RB, IL-18R1, MCP-4, CCL19, IL-17C |
| **MAPK Cascade** | AXIN1, CCL25, FGF-19, TGF-alpha, LAP TGF-beta-1, Beta-NGF, TNF, IL6, CCL3, CCL4 , MCP-1, OSM, HGF, LIF, VEGF-A, NT-3, CD40, CCL11, CCL23, CX3CL1 , CCL20, MCP-2, MCP-3, EN-RAGE , SLAMF1, MCP-4, CCL19, FGF-23, FGF21, IL5, FGF-5, IL-2RB, SCF, GDNF, Flt3L, IL-2, IL-18, ARTN, NRTN |
| **Regulation of IR** | ADA, LAP TGF-beta-1, TNFB, TNF, IFN-gamma, IL-4, IL6, IL10, CD40, IL-12B, IL-13, IL-2, IL-18R1, IL-18, CASP-8 , CCL19, PD-L1, CD8A |
| **Response to hypoxia** | SIRT2, uPA, ADA, LAP TGF-beta-1, TNFB |
| **Secretion** | IL-33, uPA, ADA, LAP TGF-beta-1, TNF, IFN-gamma, IL-1 alpha, IL5, IL6, CXCL1, CCL3, OSM, HGF, LIF, VEGF-A, IL10, CD40, IL-13, GDNF, IL-2, CX3CL1 , MCP-2, EN-RAGE , TNFRSF9, IL-17A, TSLP, CCL19, CD244, FGF-23, PD-L1 |

**Table S2: Association between chronological age and CSF or plasma inflammatory proteins in Aβ- CU individuals**

| Process | CSF R2 | Plasma R2 |
| --- | --- | --- |
| Apoptotic Process | 0.16 | 0.1 |
| Cell activation in IR | 0.15 | 0.1 |
| Cell Adhesion | 0.18 | 0.15 |
| Response to cytokine stimulus | 0.34 | 0.33 |
| Chemotaxis | 0.31 | 0.28 |
| Extracellular matrix organization | 0.09 | 0.1 |
| Inflammatory Response | 0.31 | 0.29 |
| MAPK Cascade | 0.24 | 0.21 |
| Regulation of IR | 0.09 | 0.12 |
| Response to hypoxia | 0.06 | 0.03 |
| Secretion | 0.15 | 0.17 |
| All Proteins | 0.41 | 0.42 |

This table shows how well CSF and plasma proteins from each inflammatory process predicted chronological age in the Aβ- CU group. Associations were tested using linear regression with adjustment for sex and education.

**Table S3: Proteins included in data-driven cluster and their coefficient values**

| **Variable** | **CSF** | **Plasma** |
| --- | --- | --- |
| EDUCAT | -0.12 | -0.09 |
| GENDER | 1.13 | 0.00 |
| OPG | 0.00 | 2.13 |
| ADA | -0.60 | 0.00 |
| IFN_gamma | 0.00 | -1.21 |
| IL_1alpha | -0.38 | 0.00 |
| IL6 | -0.63 | 0.00 |
| CCL3 | 2.35 | 0.00 |
| uPA | 0.00 | -0.26 |
| IL_18R1 | 0.00 | -1.02 |
| CCL19 | 0.00 | -0.72 |
| TRANCE | 0.00 | -0.44 |
| CCL25 | 1.62 | 0.00 |
| TNFSF14 | -1.17 | 0.00 |
| MCP_1 | 0.00 | -0.13 |
| CD6 | -1.18 | 0.00 |
| CASP_8 | 0.00 | 0.90 |
| CCL28 | 1.74 | 0.00 |
| PD_L1 | 0.76 | -1.18 |
| CCL4 | -0.41 | 0.00 |
| SCF | 0.92 | 0.80 |
| CXCL10 | -0.96 | 0.00 |
| MMP_1 | -0.59 | 0.00 |
| IL5 | 0.00 | -0.04 |
| OSM | 0.00 | -0.24 |
| HGF | 0.00 | 0.21 |
| IL_2RB | 0.00 | -0.34 |
| LIF | -0.17 | -0.57 |
| LIF_R | -0.68 | -0.84 |
| Flt3L | 0.25 | -0.26 |
| CCL20 | -0.46 | 0.00 |
| MCP_2 | -0.27 | 0.00 |
| MCP_3 | 0.00 | 0.02 |
| CXCL6 | 0.00 | 0.02 |
| CXCL9 | 1.92 | 2.58 |
| IL_15RA | 0.00 | 0.06 |
| IL_10RA | 0.00 | -0.11 |
| IL_17A | 0.00 | 0.26 |
| MCP_4 | 0.00 | 0.27 |
| FGF_23 | 0.04 | 0.00 |
| IL_20RA | 0.05 | 0.00 |
| NT_3 | 0.00 | -1.20 |
| NRTN | 0.00 | 0.03 |
| MMP_10 | 0.32 | 0.00 |
| AXIN1 | 0.00 | 0.25 |
| FGF_19 | -0.67 | 0.00 |
| TGF_alpha | -0.31 | 2.09 |

This table shows which proteins were selected in the CSF- and plasma-based clusters selected using LASSO regression. All proteins were available for initial model selection with the outcome of interest being chronological age in the amyloid-negative healthy control group.

**Table S4: Comparison of *InflammAGE* scores across processes and groups to Aβ- CU individuals**

| **Process** | **AB- CU-VAL** | **AB- MCI** | **AB- SCD** | **AB+ AD** | **AB+ MCI** | **Modality** |
| --- | --- | --- | --- | --- | --- | --- |
| Apoptotic Process | 0.4839 | 0.022 | 0.4839 | 0 | 0 | CSF |
| Cell activation in IR | 0.049 | 1.00E-04 | 0.1514 | 0 | 0 | CSF |
| Cell Adhesion | 0.1618 | 0.1717 | 0.4281 | 0 | 0.1618 | CSF |
| Response to cytokine stimulus | 0.5009 | 0 | 0.1454 | 0 | 0 | CSF |
| Chemotaxis | 0.0883 | 0 | 0.0715 | 0 | 0 | CSF |
| Extracellular matrix organization | 0.926 | 2.00E-04 | 0.926 | 0 | 0 | CSF |
| Inflammatory Response | 0.5246 | 0.0033 | 0.4406 | 0 | 0 | CSF |
| MAPK Cascade | 0.0641 | 0 | 0.0058 | 0 | 0 | CSF |
| Regulation of IR | 0.1098 | 0.1791 | 0.8146 | 0 | 0.0051 | CSF |
| Response to hypoxia | 1 | 0.9193 | 1 | 0 | 0.0166 | CSF |
| Secretion | 0.2689 | 3.00E-04 | 0.2065 | 0 | 0 | CSF |
| All Proteins | 0.0262 | 0 | 0.0213 | 0 | 0 | CSF |
| Apoptotic Process | 0.0014 | 0.0574 | 0.5095 | 1.00E-04 | 0.7097 | Plasma |
| Cell activation in IR | 0 | 0.378 | 0.378 | 0.0017 | 0.7654 | Plasma |
| Cell Adhesion | 0.0508 | 0.8317 | 0.8317 | 0 | 0.4253 | Plasma |
| Response to cytokine stimulus | 1 | 1 | 1 | 9.00E-04 | 1 | Plasma |
| Chemotaxis | 0.2374 | 1 | 1 | 0.0027 | 1 | Plasma |
| Extracellular matrix organization | 0.0051 | 0.6439 | 0.6737 | 0 | 0.7668 | Plasma |
| Inflammatory Response | 0.1456 | 1 | 1 | 0.0045 | 1 | Plasma |
| MAPK Cascade | 0.0112 | 0.0037 | 0.7055 | 0.0112 | 0.5804 | Plasma |
| Regulation of IR | 0.0478 | 0.8516 | 0.7478 | 0 | 0.8559 | Plasma |
| Response to hypoxia | 0.0044 | 1 | 0.3578 | 1.00E-04 | 1 | Plasma |
| Secretion | 0.0136 | 0.0036 | 0.5021 | 0 | 0.3038 | Plasma |
| All Proteins | 0.1373 | 0.658 | 1 | 0.003 | 1 | Plasma |

This table shows the P-values for comparing *InflammAGE* scores (inflammatory age – chronological age) for all groups and all inflammatory pathways to the *InflammAGE* scores in the Aβ- CU group. A significant P-value therefore means that the given group was characterized by increased inflammatory aging for the given inflammatory process compared to the Aβ- CU group.

**Table S5: Comparison of *InflammAGE* scores across processes and groups to Aβ- MCI individuals**

| Process | AB+ AD | AB+ MCI | Modality |
| --- | --- | --- | --- |
| Apoptotic Process | 9.00E-04 | 0.0469 | CSF |
| Cell activation in IR | 0.104 | 0.1514 | CSF |
| Cell Adhesion | 0.0762 | 1 | CSF |
| Response to cytokine stimulus | 0.8317 | 0.6552 | CSF |
| Chemotaxis | 0.0715 | 0.9636 | CSF |
| Extracellular matrix organization | 0 | 0.926 | CSF |
| Inflammatory Response | 0.5246 | 0.5246 | CSF |
| MAPK Cascade | 0.0641 | 0.6271 | CSF |
| Regulation of IR | 0.2878 | 0.8146 | CSF |
| Response to hypoxia | 0.0027 | 1 | CSF |
| Secretion | 0.6131 | 0.6131 | CSF |
| All Proteins | 0.018 | 0.6644 | CSF |
| Apoptotic Process | 0.5095 | 0.0574 | Plasma |
| Cell activation in IR | 0.4683 | 0.4683 | Plasma |
| Cell Adhesion | 0 | 0.8317 | Plasma |
| Response to cytokine stimulus | 0.5656 | 1 | Plasma |
| Chemotaxis | 0.2241 | 1 | Plasma |
| Extracellular matrix organization | 0.0548 | 0.6737 | Plasma |
| Inflammatory Response | 0.0994 | 1 | Plasma |
| MAPK Cascade | 0.7055 | 0.1772 | Plasma |
| Regulation of IR | 0.0074 | 0.8516 | Plasma |
| Response to hypoxia | 0.0016 | 1 | Plasma |
| Secretion | 0.5021 | 0.3038 | Plasma |
| All Proteins | 0.658 | 0.8639 | Plasma |

This table shows the P-values for comparing *InflammAGE* scores (inflammatory age – chronological age) for all groups and all inflammatory pathways to the *InflammAGE* scores in the Aβ- MCI group. A significant P-value therefore means that the given group was characterized by increased inflammatory aging for the given inflammatory process compared to the Aβ- MCI group.
